# Recognition and diagnosis of Alzheimer’s Disease using T1-weighted magnetic resonance imaging via integrating CNN and Swin vision transformer

**DOI:** 10.1016/j.clinsp.2025.100673

**Published:** 2025-06-17

**Authors:** Yanlei Wang, Hui Sheng, Xueling Wang

**Affiliations:** a Department of Radiology, Yantaishan Hospital, Yantai, 264000, Shandong, China; b Shandong University of Political Science and Law, Jinan, 250000, Shandong, China

**Keywords:** Alzheimer’s disease, Classification, Deep learning, Vision transformer, Machine vision

## Abstract

**Purpose:**

Alzheimer’s disease is a debilitating neurological disorder that requires accurate diagnosis for the most effective therapy and care.

**Methods:**

This article presents a new vision transformer model specifically created to evaluate magnetic resonance imaging data from the Alzheimer’s Disease Neuroimaging Initiative dataset in order to categorize cases of Alzheimer’s disease. Contrary to models that rely on convolutional neural networks, the vision transformer has the ability to capture large relationships between far-apart pixels in the images. The suggested architecture has shown exceptional outcomes, as its precision has emphasized its capacity to detect and distinguish significant characteristics from MRI scans, hence enabling the precise classification of Alzheimer’s disease subtypes and various stages. The model utilizes both the elements from convolutional neural network and vision transformer models to extract both local and global visual patterns, facilitating the accurate categorization of various Alzheimer’s disease classifications. We specifically focus on the term ’dementia in patients with Alzheimer’s disease’ to describe individuals who have progressed to the dementia stage as a result of AD, distinguishing them from those in earlier stages of the disease.

**Results:**

Precise categorization of Alzheimer’s disease has significant therapeutic importance, as it enables timely identification, tailored treatment strategies, disease monitoring, and prognostic assessment.

**Conclusion:**

The stated high accuracy indicates that the suggested vision transformer model has the capacity to assist healthcare providers and researchers in generating well-informed and precise evaluations of individuals with Alzheimer’s disease.

## Highlights

• This study proposes a new vision transformer to evaluate MRI from the ADNI dataset.
• The proposed model captures global relationships between long-range pixels.
• The suggested model achieves high accuracy in classifying AD classes.

## Introduction

Alzheimer’s disease (AD), a degenerative neurological ailment, has emerged as a widespread condition and is now acknowledged as the fourth leading cause of mortality in industrialized nations. The primary manifestations of AD are amnesia and cognitive deterioration, resulting from the degeneration and demise of neurons accountable for memory function.^1^ Mild cognitive impairment (MCI) is a stage that occurs between normal cognitive function and the beginning of AD.^2^ Over time, Alzheimer’s disease generally progresses from the first stages of MCI to a condition of dementia. Note that the term ’dementia in patients with Alzheimer’s disease’ is utilized throughout this study to denote patients who exhibit dementia symptoms attributable to AD, reflecting the advanced stage of the disease spectrum. Studies indicate that the yearly rate of conversion from MCI to AD exceeds 10 percent. Early detection of MCI is vital as it has the ability to hinder or delay the progression to AD.

Prior studies emphasize that early moderate cognitive impairment (EMCI) is distinguished by the earliest indications of MCI. Conversely, late mild cognitive impairment (LMCI)^3^ or progressive mild cognitive impairment (PMCI)^4^ are terms used to describe symptoms that deteriorate with time. Note that these terms were introduced from deep learning methods and not for clinical use. Healthcare practitioners must be watchful as the symptoms progress and transition through several phases. Scientists have a challenging problem when it comes to identifying variations in specific symptoms across different populations. Standardized testing protocols and imaging techniques, such as magnetic resonance imaging (MRI),^5^ positron emission tomography (PET),^6^ and computed tomography (CT),^7^ play a crucial role in the experimental procedures related to these diagnostic tools.

MRI is a well-recognized and secure diagnostic technique known for its capacity to detect many medical diseases, including neurological illnesses. The wide range of applications of MRI is attributed to its exceptional sensitivity, which allows for the prompt detection of illnesses. Each MRI sequence has unique characteristics that make them appropriate for detecting particular ailments. MRI is often the preferred imaging modality for categorizing AD. In addition, MRI images offer a variety of characteristics that are crucial for categorizing and diagnosing MCI or AD. These include assessments of grey and white matter volumes, cortical thickness, and cerebral spinal fluid (CSF) levels, as stated by Liu et al. ^8^ These measurements can aid in determining the progression of the disease. Pre-trained deep learning models have recently shown promise in automatically detecting cognitive deficits from brain MRI data. Multiple convolutional neural networks have undergone pre-training and subsequently been used for the analysis of MRI data in order to diagnose AD.^9^

Deep learning methods often need a large collection of image samples and include convolutional processes for both model training and feature extraction. Nevertheless, it is common for public AD datasets to have unbalanced class distributions, where some classes are inadequately represented. When there is a shortage of image samples available for training a convolutional neural network (CNN), conventional data augmentation techniques are often used. This study exploited the open Alzheimer’s Disease Neuroimaging Initiative (ADNI) dataset^10^ to overcome the problem of small sample numbers. In addition, several data augmentation techniques were used to increase the quantity of AD images in this investigation. These strategies generate extra images by making slight alterations to the current training samples instead of obtaining new images. This artificially boosts the number of images in the dataset.^11^ Furthermore, transfer learning was used as a technique to address the issue of class imbalance and limited sample availability, in order to improve the accuracy of image classification for AD.

**Table 1:** Details of the raw data sample distribution in this study.

Type	No. of subjects	Mean age (years)	Mean education level (years)
AD	168	75.4	15.6
LMCI	70	71.2	16.2
MCI	210	70.8	16.7
EMCI	238	69.5	16.9
HC	575	70.6	16.4

Based on the previous discussion, this study introduces both a conventional CNN model^12^ and an advanced vision transformer architecture designed to classify AD. This model utilizes a self-attention mechanism to effectively handle the vast interactions among pixels across the image. The computational requirement of self-attention increases quadratically with the image resolution. Consequently, it would be unfeasible to directly implement the original vision transformer model,^13^ particularly when dealing with high-resolution images. This would need a substantial augmentation in memory and processing capacity. In order to address this issue, the vision transformer used in this work utilizes a sliding window methodology, resulting in a substantial reduction in both the number of parameters and the computing burden. In addition, pre-trained weights are used for AD classification based on the ImageNet dataset.^14^ The performance of the proposed technique is evaluated using the ADNI dataset,^10^ which consists of several kinds of AD. Additionally, a comparative study is conducted to assess the effectiveness of the proposed approach in comparison to existing best practices. The empirical findings demonstrate that the suggested technique surpasses the currently dominant methodologies in terms of many assessment measures.

In summary, the key contributions of this research are outlined as follows:

• An integrated CNN and vision transformer pipeline with a shifted sliding window has been developed for the purpose of detecting and classifying AD.
• The suggested transformer model has two fundamental components and an attention mechanism. These elements are used to progressively process the standard and shifted sections inside an image.
• Empirical evidence substantiates that the suggested approach surpasses current cutting-edge algorithms.

## Related work

Recently, there has been a significant rise in the use of deep learning methods to categorize AD by analyzing data from several brain imaging modalities. Several research endeavors have used the abundance of data obtained from various imaging modalities to develop enhanced deep CNN and vision transformer models for the categorization of AD.

The adept use of deep CNN models in the study conducted by Singh et al. ^9^ has facilitated the categorization of six unique phases of AD. This development shows great potential in improving the accuracy of diagnosis and boosting our comprehension of the illness. The models accurately identify normal control (NC), significant memory concern (SMC), EMCI, LMCI, and AD. CNN models, including EfficientNet,^15^ MobileNet,^16^ DenseNet,^17^ Resnet,^18^ AlexNet,^19^ and InceptionV2,^20^ are utilized to classify brain MRI images. The models used to differentiate between different phases of AD attain average accuracy of 99.79%, 98.74%, 84.39%, 84.28%, 83.74%, 94.39%, and 88.28%, respectively.

Habiba et al. ^21^ used EfficientNet^15^ as their feature extraction method and a deep CNN as the classifier. They used a dataset that was available to the public, which consists of 6400 MRI images. Their suggested system utilizes a limited number of convolutional layers to effectively capture all features, hence improving the efficiency of feature learning and producing more precise and reliable outcomes. In addition, they used data augmentation to address the issue of data imbalance by increasing the size of the dataset for the minority class. In addition, they used the transfer learning technique by including EfficientNet to address the problem of overfitting.

Lately, vision transformer models have been used for the identification of AD using MRI images. The study done by Dhinagar et al. ^22^ evaluated several iterations of the vision transformer architecture for a range of neuroimaging tasks of different levels of difficulty. The focus was on gender and AD categorization using 3D brain MRI data. During their studies, two iterations of the vision transformer architecture attained an area under the curve (AUC) of 0.987 for gender classification and 0.892 for AD classification, respectively.

In addition, Akan, Alp, and Bhuiyan^23^ suggested using the visual transformer^13^ and bidirectional long short-term memory (bi-LSTM)^24^ to examine MRI images for the purpose of diagnosing AD. The vision transformer was used to extract characteristics from the MRI images and then turn them into a series of features. Afterwards, the bi-LSTM was used for sequence modeling in order to preserve the interconnections between related characteristics. The efficacy of the suggested model was evaluated for the binary categorization of individuals with AD using data from the ADNI dataset. Ultimately, the suggested approach was evaluated against various deep learning methods documented in existing literature. The suggested technique has shown exceptional performance in terms of accuracy, precision, F-score, and recall for the diagnosis of AD.

## Materials and methods

Within the domain of machine learning, a conventional classification procedure involves a series of fundamental stages, including data pre-processing, feature extraction, feature selection, and the actual classification process. These processes have been widely implemented in several applications based on artificial intelligence.

Because there is a limited amount of biological pattern data available, several classification algorithms rely on feature sets that are created manually. However, these feature sets that are created manually have inherent limitations in their ability to effectively apply to brain MRI images. Lesions often exhibit significant resemblances in terms of color, size, form, and texture, resulting in intricate linkages and a limited presence of distinctive characteristics. Hence, efforts to categorize AD using human feature-based methods are deemed unsuccessful. In contrast, deep learning algorithms has the ability to autonomously extract the most appropriate characteristics from the data. When comparing shallow and deep networks, particularly CNN models, it becomes evident that deep networks are better at revealing the essential characteristics required for precise image categorization. However, the ability to get meaningful embeddings is highly dependent on the amount of training data available, a resource that has not been properly used in previous studies.

### Dataset and data augmentation

Typically, a wide range of datasets may be used for the categorization of AD. However, specific AD datasets in comma-separated values (CSV) format are unsuitable for this particular investigation. Specialized organizations like Kaggle, the ADNI, and the open access series of imaging studies (OASIS) provide datasets that may be used for research and instructional purposes. The MRI dataset used in this study is derived from the ADNI database and consists of the MRI images used in the research. The ADNI dataset includes individuals with AD, MCI, and healthy control people. The dataset encompasses a diverse array of information, including genetic data, cognitive evaluations, and biomarkers from CSF, as well as MRI and PET scans, in addition to clinical particulars. The statistical data for the 1261 MRI samples used in this investigation is shown in Table 1. This distribution provides a substantial sample size for each group, which is crucial for detecting significant differences and trends. The mean ages and education levels provided in the table further support the representativeness of the sample across different demographic factors. To note that the largest group, health control (HC), with 575 subjects, provides a strong control base, while the smaller groups like LMCI and AD, with 70 and 168 subjects respectively, still offer a substantial sample size for comparative analyses.

Deep learning models rely heavily on extensive datasets, and their capacity to make generalizations improves as the volume of data increases. In this research, data augmentation is performed using a range of operations, including rotation, flipping, random cropping, alterations to brightness and contrast, pixel jittering, manipulation of the aspect ratio, random shearing, zooming, and vertical and horizontal shifting. Data augmentation is a technique used to artificially amplify the quantity of existing data. In addition, we have employed a balanced strategy during data augmentation to ensure that each category is equally represented in the augmented dataset. After the enhancement procedure, there are a total of 5000 MRI images. It is crucial to acknowledge that every category of MRI images consists of 1000 slices. This enhancement is achieved by integrating slightly altered copies of the current training data instead of obtaining whole new data. The objective of this strategy is to enhance the variety of the dataset by making little modifications to the current data instances or by generating synthetic data derived from the existing data. All images were downsized to a resolution of 224 × 224.

### Details of the backbone

**Fig. 1:**
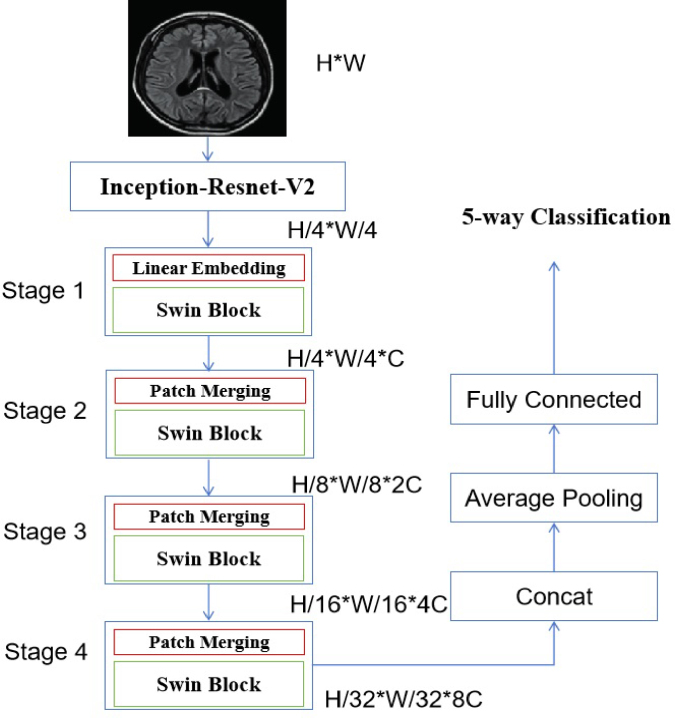
The architecture of the introduced deep learning model.

**Fig. 2:**
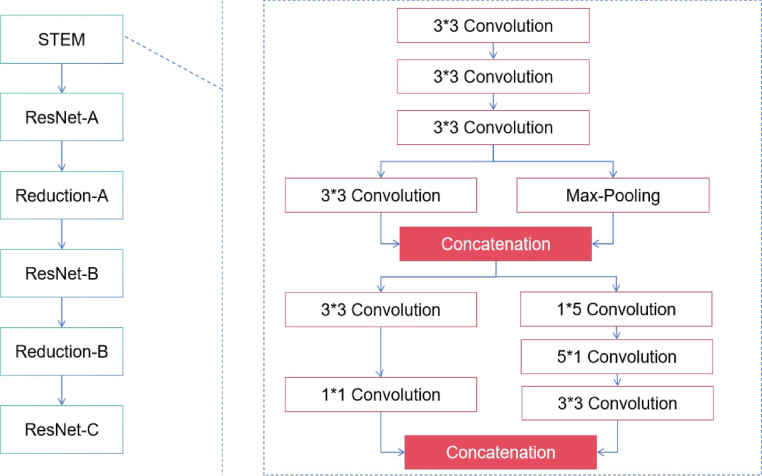
The detailed modules in the proposed Inception-Resnet-V2 model.

This study uses an integrated model of CNN and swin transformer. The proposed model includes two continuous steps. Initially, the present study utilized the Inception-Resnet-V2 architecture^12^ as the feature extractor. The diagram shows Fig. 1, Fig. 2 demonstrates that the convolutional operators and max-pooling units in the stem unit are used to extract the inner embedding of the input images.

In addition, the characteristics retrieved from the stem module were refined using the Inception-Resnet-A, Inception-Resnet-B, Inception-Resnet-C, Reduction-A, and Reduction-B modules integrated into the Inception-Resnet-V2 model. All of these modules consist of a set of convolutional operators of different sizes, namely 1 × 1, 3 × 3, 1 × 5, and 5 × 1. Furthermore, the 1 × 1 operation is intended to decrease the overall dimensions of the extracted features. Then for the input of the swin transformer, each patch having dimensions of 16 × 16. The characteristics of each patch are determined by aggregating the pixel values inside that patch, using a technique like the one used by the vision transformer.^13^

During the first stage, the original feature is projected into a certain dimension using a linear embedding layer. Subsequently, a sequence of Swin transformer blocks^25^ is used, comprising of two separate self-attention processes. Furthermore, it is said that the number of tokens in each block stays consistent, perfectly aligning with the size provided by the linear embedding layer ($\frac{H}{4}\times \frac{W}{4}$).

The suggested approach employs components to merge patches, resulting in a 50% reduction in feature size and enabling the creation of a structured representation. Stage 2 includes the first module for combining patches and transforming characteristics, which is subsequently used in Stages 3 and 4. In addition, the resolution of the output elements gradually increases from Stage 1 to Stage 4. The formulas $\frac{H}{4}\times \frac{H}{4}\times C$, $\frac{H}{8}\times \frac{H}{8}\times C$, $\frac{H}{16}\times \frac{H}{16}\times C$, and $\frac{H}{32}\times \frac{H}{32}\times C$ represent a series of calculations involving the variables H and C. The key differentiating factor between the Swin vision transformer, as suggested in the work of Liu et al.,^25^ and the original vision transformer introduced by Dosovitskiy et al.,^13^ is the hierarchical representation. This differentiation is accomplished by collectively implementing Stage 2, Stage 3, and Stage 4. In summary, the output vector is produced by using global average pooling in combination with a fully-connected layer. The output vector’s size is determined by the equation $N=\frac{H}{32}\times \frac{W}{32}$. The linear classifier considers just the top C components of the output vector.

### Swin transformer block

**Fig. 3:**
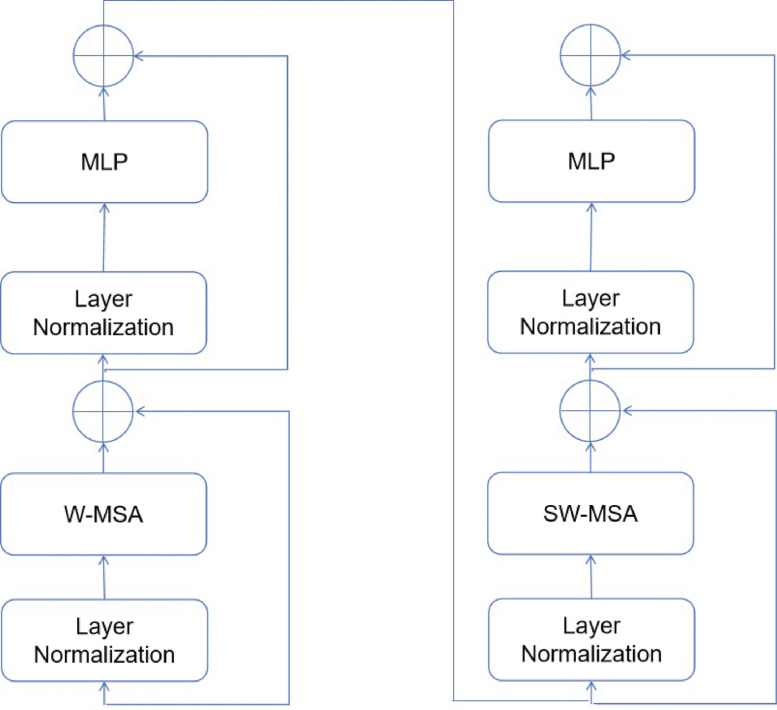
The constituents of the proposed vision transformer model. The acronyms W-MSA and SW-MSA represent the multi-head self-attention modules that include standard and shifted windows, respectively. MLP is an abbreviation for multilayer perceptron.

As shown in Fig. 1, each stage consists of a set of Swin transformer blocks, as demonstrated in Fig. 3, where each block is comprised of two successive Swin transformer modules. The W-MSA and SW-MSA modules represent the multi-head self-attention (MSA) technique, which is implemented using a conventional window method and a shifted window approach, respectively.

The subsequent mathematical equations can be utilized to express the sequential Swin Transformer modules’ numerical formulation.

(1) $${\overset{ˆ}{z}}^{l}=W-MSA\left(LN\left(z^{l-1}\right)\right)+z^{l-1},$$

(2) $$z^{l}=MLP\left(LN\left({\overset{ˆ}{z}}^{l}\right)\right)+\overset{ˆ}{z},$$

(3) $${\overset{ˆ}{z}}^{l+1}=SW-MSA\left(LN\left(z^{l}\right)\right)+z^{l},$$

(4) $$z^{l+1}=MLP\left(LN\left({\overset{ˆ}{z}}^{l+1}\right)\right)+{\overset{ˆ}{z}}^{l+1},$$

W-MSA stands for window-based multi-head self-attention mechanism; MLP is an abbreviation for multi-layer perceptron as described in Tolstikhin’s research on MLPMixer,^26^ SW-MSA refers to the shifted-window multi-head self-attention approach; and LN is short for layer normalization, a technique explained in Ba’s study on layer normalization.^27^

### Shifted window mechanism

The MSA module, based on a window-based process, differs from the original vision transformer that depended on global self-attention. The latter necessitated the computation of the interactions between each individual token and the whole collection of tokens. The MSA module, which is based on windows, functions inside a window of size M×M, usually with M being set to 7. This approach reduces the computing workload by reducing the quantity of data that has to be processed. Hence, the inclusion of the window-based self-attention mechanism leads to a more manageable computational complexity compared to the quadratic complexity of the vision transformer,^13^ which increases with the image dimensions h×w.

(5) $$\Omega \left(MSA\right)=4hwC2+2{\left(hw\right)}^{2}C,$$

(6) $$\Omega \left(W-MSA\right)=4hwC^{2}+2M^{2}hwC,$$

**Fig. 4:**
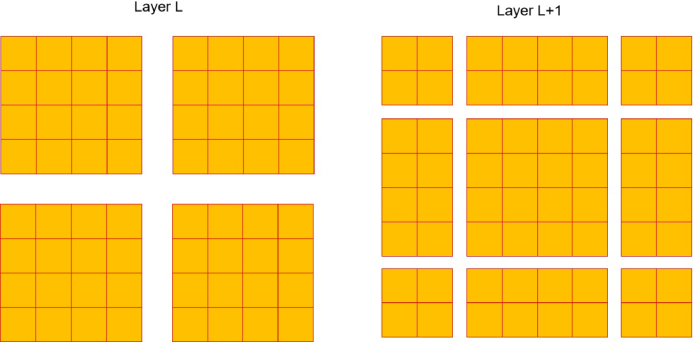
The procedure of the SW-MSA mechanism employed in the proposed methodology.

Moreover, the SW-MSA technique is specifically developed to enhance the encoding of the comprehensive connections between pixels inside every window. Using SW-MSA enables the optimization of the interaction among several windows. The method of dividing a conventional window into sections may be seen in layer l, as shown in Fig. 4. Self-attention is computed for each individual window. In the subsequent layer, the window partitioning is adjusted both horizontally and vertically, leading to a wider range of window options. Consequently, the calculation of self-attention in Layer l requires the manipulation of the windows that existed in Layer l.

The loss function used in the proposed model is the cross-entropy loss, which is of significant importance to note. The loss is calculated by comparing the true category of the image with the classification result generated by the proposed approach, as shown in Fig. 1.

### Transfer learning

Transfer learning is a technique in machine learning where an algorithm is first trained on one dataset and then applied to another dataset, which is a job that has similarities with the original one. This method is often referred to as domain adaptation and transfer learning. They are used to facilitate the process of generalizing to a new setting. This method is very successful in deep neural networks, irrespective of the substantial data and resource requirements. The current datasets often lack the necessary diversity and include a limited number of images, making them inadequate for training deep neural networks from the beginning. Transfer learning is seen as a feasible remedy for this problem.^28^

This research uses transfer learning to categorize images into eight distinct categories associated with AD. The pre-trained models used in this study are sourced from ImageNet, a dataset originally employed for the classification of a diverse range of 1000 objects.^14^ In order to properly utilize transfer learning, it is necessary to adhere to three essential protocols. At first, a sequential adjustment is made to the last trainable layer of each neural network in order to facilitate the recognition of AD images. Furthermore, the weights and biases of the preceding layers are adjusted to preserve their capacity to extract fundamental characteristics. Ultimately, to improve the learning process for the fundamental layers, one might augment the learning rate coefficients for both the weights and biases.

The authors conducted this diagnostic study following STARD guidelines.^29^

## Experimental results

### Implementation details

The tests were conducted using two NVIDIA RTX 3080 GPUs and the PyTorch framework.^30^ The chosen backbone architecture for the created model is the Swin-T vision transformer.^25^ The input images were uniformly scaled to a resolution of 224 pixels in both width and height. In addition, the authors used the pre-trained weights from ImageNet^14^ to initialize the suggested vision transformer. The researchers selected a batch size of 16, used the Adam Optimizer for the hyperparameter, established a learning rate of 1e-5, determined a depth of 16, and set the number of epochs to 200. In order to guarantee the accuracy and dependability of the findings, a 10-fold cross-validation technique was used in the comparison studies. Normally, 90% of the MRI samples were assigned to the training set, while the remaining 10% were assigned to the test set.

The following equations define the metrics of accuracy, precision, recall, and F1 score, which are used to evaluate the performance of the proposed model and other techniques.

(7) $$Accuracy=\frac{\left(TP+TN\right)}{\left(TP+TN+FP+FN\right)},$$

(8) $$Precision=\frac{TP}{TP+FP},$$

(9) $$Recall=\frac{TP}{TP+FN},$$

(10) $$F1_{score}=\frac{2\times Precision\times Recall}{Precision+Recall,}$$

In these equations, the terms TP, TN, FP, and FN represent the counts for true positives, true negatives, false positives, and false negatives, respectively.

### Ablation study

In order to validate the efficacy of the recently implemented vision transformer, a series of ablation tests were carried out. These investigations included assessing the suggested models using other arrangements, diverging from the original setup that was executed. As part of the ablation study, we conducted tests on the Swin Transformer block using three alternative combinations of the W-MSA and SW-MSA modules. The tested combinations included two iterations of the W-MSA module, two iterations of the SW-MSA module, and a fusion of both W-MSA and SW-MSA modules. The findings were achieved by applying three combinations, as shown in Tables Table 2, Table 3, and Table 4, to 30

Tables Table 2, Table 3, and Table 4 demonstrate that the suggested technique produces superior outcomes when using the optimal settings. When the transformer model was deployed on a subset that comprises 30% of the dataset, it demonstrated superior performance compared to the other options. Consequently, this model was selected as the fundamental framework for further research.

**Table 2:** Outcome of the proposed model containing two continuous instances of W-MSA modules.

Combination	Accuracy (%)	Precision (%)	Recall (%)	F1 score (%)
AD	98.22	98.67	99.96	99.35
LMCI	96.22	95.42	96.18	95.38
MCI	99.44	99.25	98.72	99.06
EMCI	98.67	97.91	98.54	97.81
HC	95.33	94.33	96.29	98.47
Average	97.58	97.12	97.94	98.01

**Table 3:** Outcome of the proposed model containing two continuous instances of SW-MSA modules.

Combination	Accuracy (%)	Precision (%)	Recall (%)	F1 score (%)
AD	98.56	98.62	99.83	99.28
LMCI	96.33	96.14	96.35	96.25
MCI	99.11	99.18	98.90	99.42
EMCI	98.33	97.96	98.82	98.13
HC	96.11	94.36	96.15	97.58
Average	97.69	97.25	98.01	98.13

**Table 4:** Outcome of the proposed model containing a mixture of both the W-MSA and SW-MSA modules.

Combination	Accuracy (%)	Precision (%)	Recall (%)	F1 score (%)
AD	98.67	98.75	99.91	99.21
LMCI	96.44	97.23	96.63	96.34
MCI	99.08	99.26	98.87	99.51
EMCI	98.61	98.57	98.79	98.25
HC	96.11	94.84	96.52	98.02
Average	97.78	97.73	98.14	98.27

### Experimental results

The results of training the suggested approach on the whole training set are first provided in Table Table 5.

**Fig. 5:**
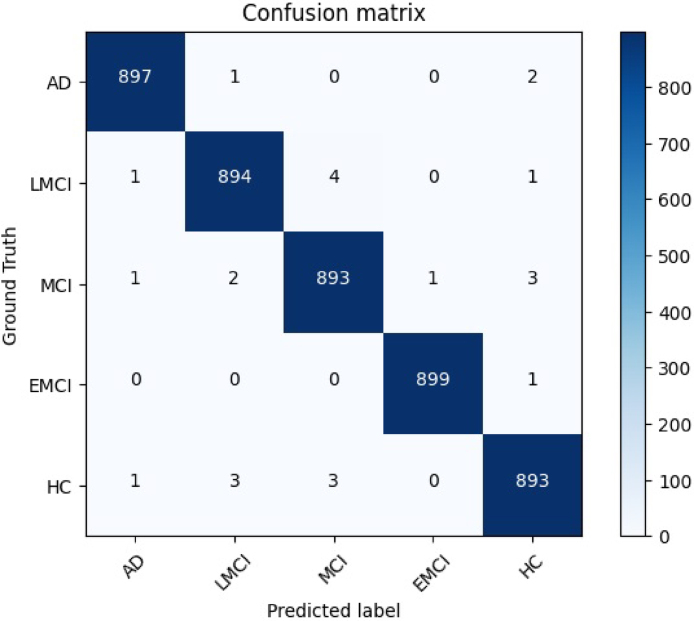
The confusion matrix pertaining to the proposed technique on the leveraged dataset prior to employing transfer learning mechanism.

**Fig. 6:**
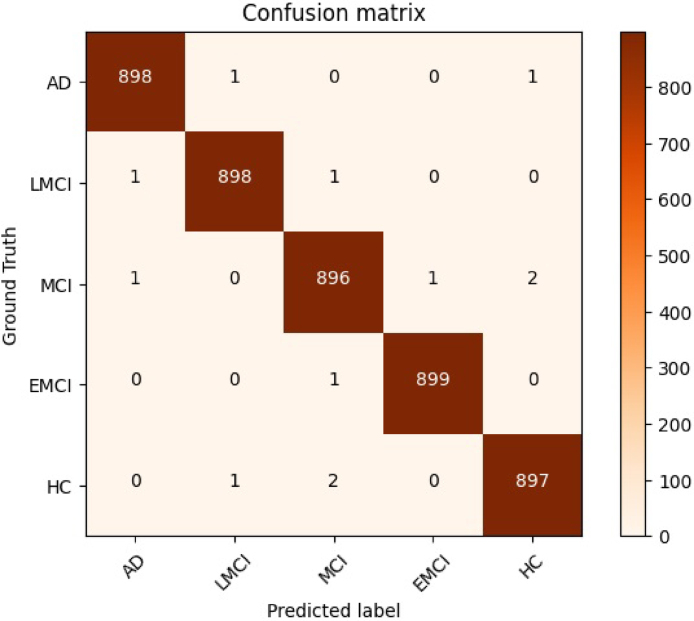
The confusion matrix pertaining to the proposed technique on the leveraged dataset after using the transfer learning mechanism.

Additionally, Fig. 5, Fig. 6 demonstrate the effectiveness of the suggested strategy both before and after the implementation of transfer learning. The use of transfer learning has undeniably contributed to the improvement of the performance of the suggested strategy.

**Table 5:** Outcome of the proposed model implemented on the entire training set.

Combination	Accuracy (%)	Precision (%)	Recall (%)	F1 score (%)
AD	99.67	98.82	99.97	99.83
LMCI	99.33	97.47	97.79	97.52
MCI	99.22	99.33	99.12	99.62
EMCI	99.89	98.41	98.75	99.05
HC	99.22	94.92	97.01	98.33
Average	99.47	97.79	98.53	98.87

In order to evaluate the performance of the proposed method fairly, we conducted comparative experiments with state-of-the-art techniques in the field.[31], [32], [33], [34], [35], [36], [37], [38], [39], [40] The comparison tests were especially targeted at the categorization task.

According to the data shown in Table Table 6, the suggested classification approach demonstrates superior accuracy compared to the majority of other competing methods. The research^40^ demonstrates a higher accuracy rate of 99.57% compared to the recommended method’s accuracy rate of 99.45%. Nevertheless, the study conducted by Ref. 40 employs a 4-way classification, while the suggested technique is specifically built for a 5-way classification problem. Ultimately, the suggested approach surpasses CNN-based deep learning algorithms, thereby emphasizing its ability to effectively capture intricate connections among pixels over wider image regions.

**Table 6:** Comparison performance between the state-of-the-arts and the proposed approach (6-way includes AD/LMCI/MCI/EMCI/HC/SMC, 5-way contains AD/LMCI/MCI/EMCI/HC, 4-way consists of AD/LMCI/EMCI/HC, 3-way denotes AD/MCI/HC, and 2-way classification represents AD/HC).

Method	Dataset	Model	Category	Accuracy (%)
Parmar et al. ^31^	ADNI	CNN	4-way	93.00
Fu’adah et al. ^32^	ADNI	AlexNet	4-way	95.00
Murugan et al. ^33^	ADNI	CNN	4-way	95.23
Celebi et al. ^34^	ADNI	Xception	3-way	95.81
Noh et al. ^35^	ADNI	CNN-LSTM	4-way	96.43
Buvaneswari et al. ^36^	ADNI	GoogLeNet	2-way	97.15
Ramzan et al. ^37^	ADNI	Resnet 18	5-way	97.88
Akter et al. ^38^	ADNI	Inception V3	6-way	98.68
Odusami et al. ^39^	ADNI	Resnet 18-DenseNet	5-way	98.86
EI-Assy et al. ^40^	ADNI	CNN	4-way	99.57
Our work	ADNI	Vision Transformer	5-way	99.47

### Discussion

CNN-based deep learning models excel in extracting feature maps from images by utilizing their convolutional layers to detect patterns and structures. It is widely believed that increasing the depth of these network topologies has the potential to enhance their ability to extract features. Nevertheless, the efficiency of CNNs may be limited by their emphasis on small receptive fields inside images. Although this approach is advantageous for most tasks, it may fail to encompass the wider context or long-range relationships between distant pixels. This constraint is partially responsible for the remarkable performance of CNNs in tasks where local characteristics are able to differentiate.

However, expanding to more complex CNN models frequently necessitates a corresponding increase in processing resources, which may be a considerable obstacle. When it comes to visual depictions of AD, the regions affected by lesions are usually distributed across the whole image rather than being limited to a particular location. Mere augmentation of layers in CNN models does not guarantee enhanced classification performance, particularly in cases involving images where the local receptive field may not adequately include the global context.

In order to tackle these difficulties, this research presents a model for image categorization that utilizes a vision transformer-based approach. The objective is to make use of the extensive connections between pixels. The suggested method effectively captures the relationships between distinct areas of a image by using a Multi-Scale Attention (MSA) mechanism. This enables the model to retain the important contextual information necessary for precise categorization. The Swin vision transformer, an enhanced version of the conventional vision transformer, is renowned for its capacity to extract significant characteristics from images while also demonstrating greater computing efficiency. This is accomplished by using a hierarchical framework and shifting windows in the self-attention mechanism, allowing it to effectively analyze visuals in a non-local way.

Nevertheless, the research did have several drawbacks. A significant limitation was the inconsistency and fluctuation in the quality and uniformity of the image samples in the dataset utilized for experiments. This lack of consistency may have adversely affected the model’s capacity to generalize successfully. In spite of the efficiency improvements of the Swin vision transformer, models based on vision transformers still require significant computational resources to achieve optimal performance. This can restrict their accessibility and practicality, particularly for large-scale applications or in environments with limited resources.

## Conclusion

The main objective of this study is to create a categorization system for AD images by using a network structure that exploits the capabilities of vision transformers. This strategy has shown exceptional performance in comparison to current state-of-the-art techniques. The suggested model’s performance is substantiated by empirical data derived from a comprehensive dataset, confirming its ability to precise categorize AD images.

Vision transformers have emerged as a notable breakthrough in the area of machine vision, demonstrating potential in tackling problems that typical CNNs may struggle with, thanks to their innate capability to capture global relationships within images. The efficacy of the suggested paradigm in this research serves as evidence of the revolutionary influence that vision transformers may have on the domain. In the future, the advancements made using vision transformers are anticipated to stimulate more research and development in the field of machine vision. There is expected to be an increasing focus on investigating multi-modal and multi-label deep learning models. The objective of these models is to enhance the accuracy of AD categorization and prediction by integrating various forms of data and effectively managing many labels per image, respectively.

A potential avenue for future study might be the use of supplementary imaging techniques, such as functional MRI or positron emission tomography, in conjunction with structural MRI to provide a more comprehensive understanding of the illness. In addition, the investigation of multi-label classification frameworks will allow for the simultaneous detection of different stages or subtypes of AD within a single image, hence improving the diagnostic capabilities of the models. Moreover, the ongoing enhancement in transformer architectures, including the integration of more advanced attention mechanisms or the creation of hybrid models that leverage the advantages of CNNs and transformers, will have a pivotal impact on expanding the limits of what can be accomplished in AD classification and prediction.

## CRediT authorship contribution statement

**Yanlei Wang:** Visualization, Software, Validation, Writing – original draft, Writing – review & Editing. **Hui Sheng:** Visualization, Investigation, Software, Validation, Writing – original draft, Writing – review & Editing. **Xueling Wang:** Conceptualization, Methodology, Software, Data curation, Writing – original draft, Writing – review & editing.

## Ethics statement

The study protocol of this work has been reviewed and approved by the Ethics Committee of Yantaishan Hospital, with the protocol number 2024-01071. This approval ensures that our study adheres to the highest ethical standards and complies with all relevant regulations and guidelines.

## Declaration of competing interest

The authors declare that they have no known competing financial interests or personal relationships that could have appeared to influence the work reported in this paper.

## Data Availability

The ADNI dataset used in this study can be downloaded from https://adni.loni.usc.edu/.
